# IGF-IR cooperates with ERα to inhibit breast cancer cell aggressiveness by regulating the expression and localisation of ECM molecules

**DOI:** 10.1038/srep40138

**Published:** 2017-01-12

**Authors:** Nikolaos A. Afratis, Panagiotis Bouris, Spyros S. Skandalis, Hinke A. Multhaupt, John R. Couchman, Achilleas D. Theocharis, Nikos K. Karamanos

**Affiliations:** 1 Biochemistry, Biochemical Analysis & Matrix Pathobiology Res. Group, Laboratory of Biochemistry, Department of Chemistry, University of Patras, Patras 26110, Greece; 2 Biotech Research and Innovation Center, University of Copenhagen, Ole Maaløes Vej 5, 2200 Copenhagen N, Denmark

## Abstract

IGF-IR is highly associated with the behaviour of breast cancer cells. In ERα-positive breast cancer, IGF-IR is present at high levels. In clinical practice, prolonged treatment with anti-estrogen agents results in resistance to the therapy with activation of alternative signaling pathways. Receptor Tyrosine Kinases, and especially IGF-IR, have crucial roles in these processes. Here, we report a nodal role of IGF-IR in the regulation of ERα-positive breast cancer cell aggressiveness and the regulation of expression levels of several extracellular matrix molecules. In particular, activation of IGF-IR, but not EGFR, in MCF-7 breast cancer cells results in the reduction of specific matrix metalloproteinases and their inhibitors. In contrast, IGF-IR inhibition leads to the depletion by endocytosis of syndecan-4. Global important changes in cell adhesion receptors, which include integrins and syndecan-4 triggered by IGF-IR inhibition, regulate adhesion and invasion. Cell function assays that were performed in MCF-7 cells as well as their ERα-suppressed counterparts indicate that ER status is a major determinant of IGF-IR regulatory role on cell adhesion and invasion. The strong inhibitory role of IGF-IR on breast cancer cells aggressiveness for which E2-ERα signaling pathway seems to be essential, highlights IGF-IR as a major molecular target for novel therapeutic strategies.

Breast cancer is the most common type of cancer among women^1^. Steroid hormones and their receptors are of high significance in breast cancer since many tumours are hormone-dependent and they are often correlated with high mortality rates^2^. Estrogen receptors (ERs) are significant regulators of many vital processes of breast cancer cells. Due to their significance in breast cancer biology, ER status classifies breast tumors in two categories: ER-positive (luminal A and B) and ER-negative (normal-like, HER-2 enriched, basal and claudin-low)^3^. ERs exist in two main forms: ERα and ERβ. However, due to the fact that two-thirds of breast tumors are ERα positive, most studies evaluate the role of this particular receptor in disease progression.

IGF-IR is a receptor tyrosine kinase of high significance in breast cancer. Its activation plays pivotal roles in cell proliferation and differentiation, as well as in cell-cell adhesion. Several studies indicate a correlation between ERα and IGF-IR activities^4^,^5^. More specifically, in a non-genomic process, E2 induces the interactions of membrane ERs with several proteins, such as growth factor-dependent kinases or adaptor proteins. A portion of ERs has the ability to localize at the membrane in multiprotein complexes. Thus their activation by E2 triggers the initiation of several downstream signaling molecules, such as c-Src, the regulatory subunit of PI-3K (p85), MAPK, AKT, p21ras and PKC^6^. This response pathway is very rapid compared to the genomic pathway. In addition, the non-genomic pathway may affect several cell functions including proliferation, survival and apoptosis^7^. It has been reported that the binding of E2 to membrane ERs triggers the rapid activation of growth factor receptors such as IGF-IR and EGFR and their downstream signaling pathways^8^,^9^,^10^,^11^,^12^. This cross-talk between growth factor receptors and ERs may also regulate breast cancer cell growth^13^ as well as the expression of extracellular matrix (ECM) macromolecules^14^.

ECM is a highly dynamic and functional network, which consists of a variety of molecules including collagens, glycoproteins, matrix proteinases and proteoglycans (PGs). This network creates the scaffold for tissue and organ establishment. Changes in the expression of ECM molecules as well as compositional alterations among them markedly affect the assembly of ECM and its ability to regulate many crucial cellular functions^15^. ECM remodeling significantly contributes to cancer progression and development. Matrix metalloproteinases (MMPs) comprise a large family of zinc-binding endopeptidases, which together with their endogenous inhibitors (TIMPs), are highly involved in these processes. Cell migration, invasion, metastasis and angiogenesis are four integral processes in tumor development that are dependent on the surrounding microenvironment. Through their proteolytic action, MMPs degrade a variety of ECM and cell adhesion molecules, thus modulating cell–cell and cell–ECM interactions^16^,^17^.

PGs and especially cell-associated heparan sulfate PGs (HSPGs), such as syndecans and glypicans, have important regulatory roles in breast cancer cell behaviour. Alterations in HSPGs expression levels during malignancies associate with disease progression^18^. For example, elevated syndecan-1 levels, particularly in the tumour stroma, indicate poor prognosis^19^,^20^,^21^. HSPGs interact with other cell surface receptors, such as growth factor tyrosine kinase receptors and integrins. In recent studies, it has been shown that syndecan-1 regulates VE-cadherin and VEGF-mediated activation of ανβ3 integrin and, via IGF-IR, induce cell proliferation in metastatic breast cancer cells^22^,^23^,^24^,^25^. Moreover, syndecan-2 and syndecan-4 expression levels and their cross talk with EGFR and IGF-IR signaling pathways have been investigated. In ERα-positive breast cancer cells, expression levels of syndecan-2 are controlled through the EGFR signaling pathway, in contrast to syndecan-4 where the expression is regulated by IGF-IR signaling. The down-regulated levels of syndecan-2 and -4 seem to be associated with higher migratory ability of breast cancer cells^14^,^26^.

The goal of our study was to investigate the role of IGF-IR in the aggressiveness of ERα-positive breast cancer cells. We evaluated the effect of IGF-R and its crosstalk with ERα and EGFR on critical cell properties as well as on the expression and/or localisation of certain syndecans, MMPs and TIMPs in breast cancer cells. Moreover, we evaluated whether the modified levels of syndecan-4 caused by IGF-IR depletion affects breast cancer cell behaviour. Finally, in order to investigate the significance of ERα on breast cancer and the importance of the synergistic actions of IGF-IR and ERα, cell function assays on MCF-7 (ERα-positive) and MCF-7/SP10+ (ERα-suppressed) cells were evaluated.

## Results

### IGF-IR activation down-regulates the gene expression levels of specific MMPs/TIMPs

**Figure 1:**
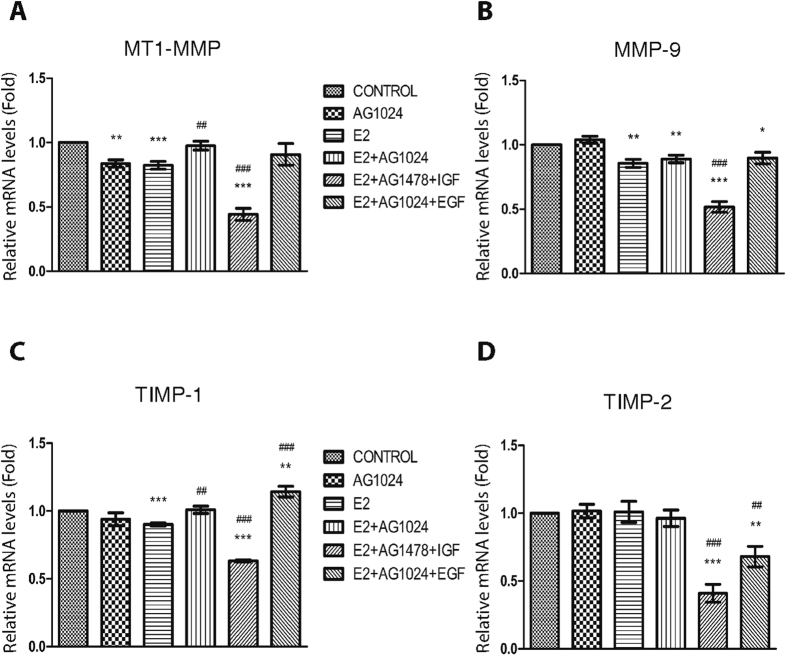
Evaluation of gene expression levels of MMPs and TIMPs after treatment with specific tyrosine kinase inhibitors and E2 in MCF-7 breast cancer cells. The effect of IGF-IR or EGFR inhibition on the constitutive and the E2-mediated gene expression of (**A**) MT1-MMP, (**B**) MMP-9, (**C**) TIMP-1, and (**D**) TIMP-2 in MCF-7 breast cancer cells. The mRNA levels were assessed by Real Time PCR analysis. Cells were pre-treated with inhibitor of EGFR (AG1478, 1 μΜ) or IGF-IR (AG1024, 1 μΜ) for 30 min, followed by the introduction of E2 (10 nM), EGF (5 ng/mL) and IGF (15 ng/mL) where indicated. Total incubation time was 16 h. Statistically significant differences compared with control or E2-treated cells are shown with *(p < 0.05), **(p < 0.01), ***(p < 0.001) or ^##^(p < 0.01), ^###^(p < 0.001), respectively.

To study the importance of matrix effectors in ERα-positive breast cancer cell behavior, we first examined the effect of ERα/IGF-IR/EGFR cross-talk on the gene expression of certain MMPs and TIMPs in MCF-7 cells. Treatment with the specific IGF-IR inhibitor, AG1024, revealed a slight down-regulation (*ca* 20–30%) of MT1-MMP expression, whereas no significant effect was observed for MMP-9, TIMP-1, and TIMP-2 (Fig. 1). Incubation with E2 also resulted in a similar slight down-regulation of MT1-MMP, MMP-9, and TIMP-1 compared to control cells. Activation of IGF-IR by IGF with concurrent EGFR inhibition in E2-treated cells resulted in a significant down-regulation (*ca* 40–60%) of all proteolytic enzymes and TIMPs examined (i.e. MT1-MMP, MMP-9, TIMP-1, and TIMP-2) (Fig. 1). On the other hand, no significant alterations were observed when EGFR was activated by EGF except for TIMP-2, which exhibited a strong down-regulation. These data indicated that IGF-IR, one of the major receptor tyrosine kinases (RTKs) in ERα-positive MCF-7 breast cancer cells, may be a key regulator of the proteolytic potential of MCF-7 cells since IGF-IR activation down-regulated MMPs/TIMPs expression that may limit tumour cell aggressiveness.

### IGF-IR inhibition down-regulates cell surface expression levels of syndecan-4

**Figure 2:**
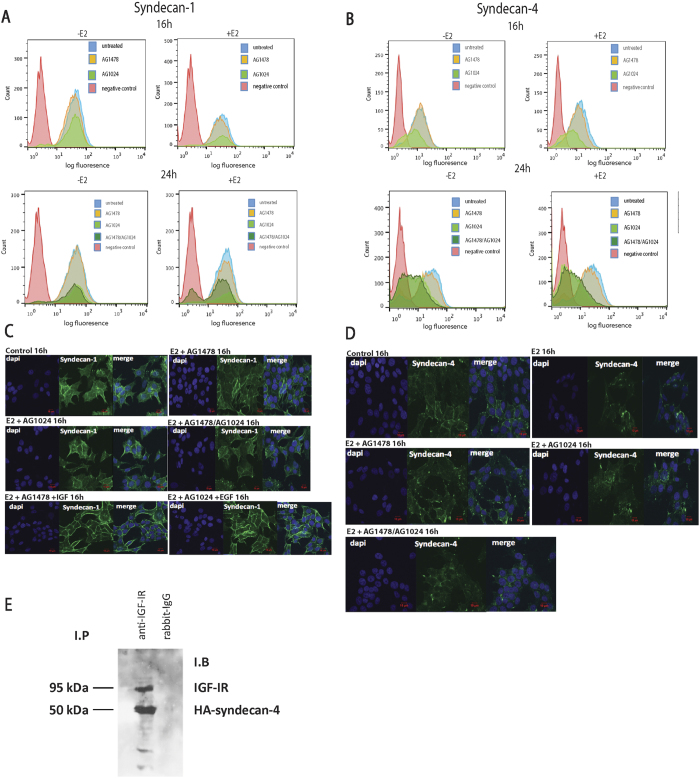
Investigation of IGF-IR and EGFR inhibition on syndecan expression levels and localisation. Evaluation of (**A**) syndecan-1 and (**B**) syndecan-4 cell surface protein levels by flow cytometry. The experiments were performed following treatments with EGFR and IGF-IR inhibitors, in the presence or the absence of E2. Immunocytochemical localisation of (**C**) syndecan-1 and (**D**) syndecan-4 after treatment with EGFR and IGF-IR inhibitors, in the presence or the absence of E2. Bars = 25 μm. (**E**) Immunoprecipitation of syndecan-4 with IGF-IR from MCF-7 cell lysates.

The well-described importance of cell membrane HSPGs in cell functional properties (such as adhesion, invasion, proliferation) as well as in cancer progression prompted us to further investigate their roles, in particular those of syndecans, in breast cancer cell behavior. Previous studies in our lab have demonstrated that specific RTKs (IGF-IR and EGFR) are key mediators for the expression of syndecans in MCF-7 cells^14^. In the present study, these observations were further investigated by performing FACS analysis and immunofluorescence microscopy. Specifically, FACS analysis showed no difference in cell surface levels of syndecan-1 after treatment with either the EGFR or IGF-IR inhibitors (AG1478 or AG1024, respectively) in the absence or presence (16 h) of E2 (Fig. 2A). Simultaneous treatment with both inhibitors in the presence of E2, led to a reduction of surface levels of a pool of syndecan-1, which was evident at 24 h (Fig. 2A). In contrast, syndecan-4 cell surface levels were significantly reduced (*ca* 50%) after treatment with IGF-IR inhibitor (AG1024) either in the absence or presence (16 h) of E2. The same results were obtained when cells were treated with both RTK inhibitors in the absence or presence (24 h) of E2 (Fig. 2B). These results indicated that IGF-IR inhibition significantly reduced cell surface syndecan-4 in an E2-independent manner, but had a little or no effect on syndecan-1. Immunofluorescence analysis showed that inhibition of either IGF-IR alone or both receptors in the presence of E2 (i.e. E2 + AG1478 + AG1024) resulted in different localization and cellular distribution of syndecan-1 and syndecan-4 characterized by a decrease of their cell surface expression, especially in the case of syndecan-4, together with stronger cytoplasmic staining (Fig. 2C and D). Moreover, treatment of cells with IGF or EGF in the presence of EGFR and IGF-IR inhibitors, respectively, resulted in restoration of cell surface levels of syndecan-1 (Fig. 2C). Taken together, these results indicated that IGF-IR regulates mainly syndecan-4, and to a lesser extent syndecan-1, expression and localization in ERα-positive MCF-7 breast cancer cells. Co-immunoprecipitation experiments revealed the presence of syndecan-4/IGF-IR complexes in syndecan-4-overexpressing cells (Fig. 2E), further indicating the cooperation of IGF-IR with syndecan-4 to accomplish cellular functions.

### IGF-IR inhibition triggers syndecan-4 endocytosis

**Figure 3:**
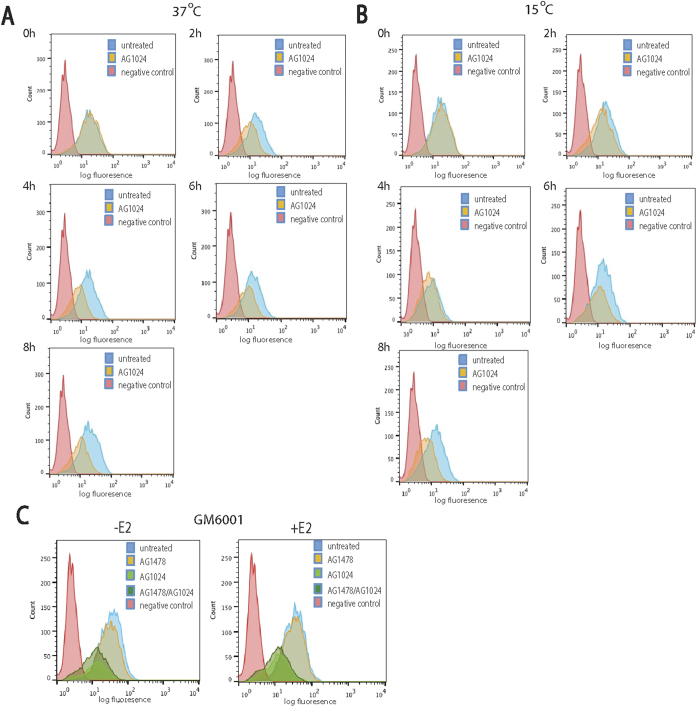
Loss of syndecan-4 on ERα+ breast cancer cells resulting from IGF-IR inhibition is reduced at low temperature but not after MMP inhibition. Time course of IGF-IR inhibition on cell surface syndecan-4 levels, in the presence or absence of E2, at 37 °C (**A**) or 15 °C (**B**). Control MCF-7 cultures were not exposed to AG1024 IGF-IR inhibitor. Lowering temperature to slow down internalisation reduced the loss of syndecan-4. (**C**) Similar FACS analysis of MCF-7 cells untreated or treated with IGF-IR inhibitor and/or EGF inhibitor in the presence or absence of E2 and the GM6001 general MMP inhibitor. Syndecan-4 levels were decreased despite the presence of sheddase inhibitor.

To further investigate the reduction of cell surface levels of syndecan-4 after 16 h of IGF-IR inhibition, FACS analyses were performed at early time points (0, 2, 4, 6 and 8 h). The results revealed that the depletion of syndecan-4 from the cell surface was evident even at 2 h after IGF-IR inhibition (Fig. 3A), which gradually increased to approx. 50% by 16 h (Fig. 2B). To examine whether the observed reduction of cell surface syndecan-4 levels after the IGF-IR inhibition was due to proteoglycan endocytosis, the same experiment was performed at 15 °C. Under these conditions, the cell surface levels of syndecan-4 remained unaltered during the first 6 h after treatment with AG1024, while a slight reduction was observed after 8 h (Fig. 3B). These results indicated that the significant reduction of cell surface levels of syndecan-4 following inhibition of IGF-IR is most likely due to syndecan-4 endocytosis. To exclude the possibility that the reduction of cell surface syndecan-4 resulted from syndecan-4 shedding, FACS analysis was performed in the presence of the metalloproteinases inhibitor GM6001. The results revealed that cell surface levels of syndecan-4 were reduced after IGF-IR inhibition regardless of the presence or absence of GM6001, demonstrating that the observed reduction was due to syndecan-4 endocytosis rather than shedding (Fig. 3C).

### Integrin-based adhesive ability of ERα+ breast cancer cells, but not the invasiveness, is regulated by IGF-IR inhibition

**Figure 4:**
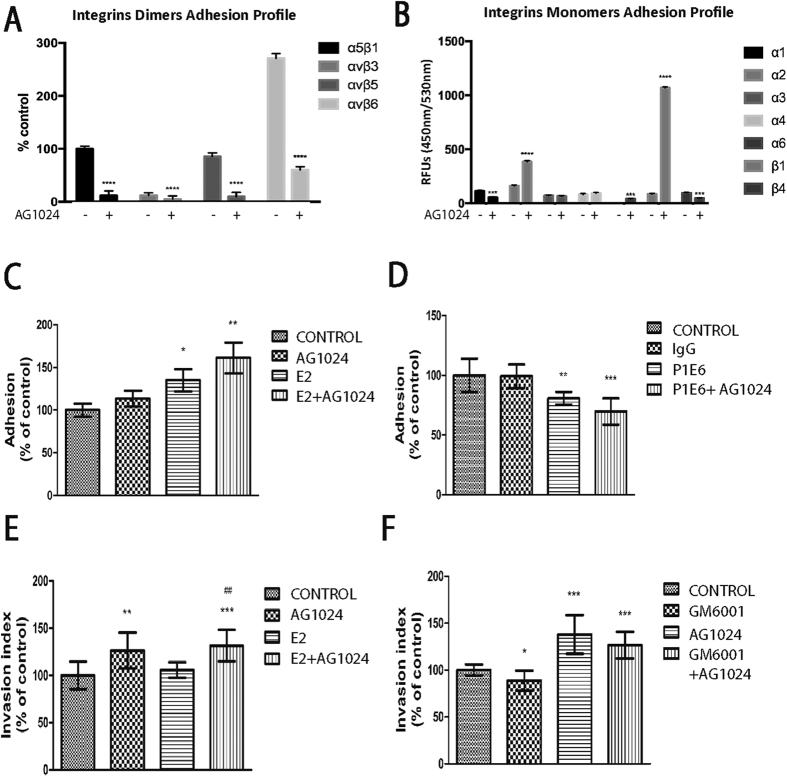
The role of IGF-IR on cell functions of MCF-7 breast cancer cells. (**A**,**B**) Evaluation of MCF-7 cell adhesion to substrates coated with integrin-specific antibodies. Treatment with IGF-IR inhibitor markedly reduced adhesion to all four substrates comprised of antibodies to “RGD-binding” integrins. In contrast, adhesion to antibodies against the collagen-binding α2 subunit was increased. Data in A normalised to α5β1 antibody adhesion in the absence of inhibitor. (**C**) The effect of IGF-IR on both the constitutive and the E2-mediated cell adhesion to type I collagen substrates by MCF-7 breast cancer cells. Adhesion was increased by E2 treatment, particularly where IGF-IR was inhibited. (**D**) The blocking of α2 integrin reduced the adhesion ability on type I collagen substrates. The inhibition with both AG1024 and anti-α2 antibody led to the enhancement of this effect. (**E**) The effect of IGF-IR inhibition on type I collagen gel invasion by MCF-7 breast cancer cells, in the presence or absence of E2. Cells were incubated with AG1024 (inhibitor of IGF-IR) (1 μΜ) for 30 min, followed by the introduction of E2 (10 nm), where indicated. Total incubation time was 24 h. Invasion was increased following IGF-IR inhibition. (**F**) Treatment of MCF-7 cell with GM6001 (MMPs inhibitor) had no effect on invasion ability of these cells. Statistically significant differences compared with control or E2-treated cells are shown as *(p < 0.05), **(p < 0.01), ***(p < 0.001) or ^#^(p < 0.05), ^##^(p < 0.01), respectively.

Given the crucial role of syndecan-4 in cell adhesion and migration^27^,^28^, we further investigated the impact of the observed syndecan-4 endocytosis induced by IGF-IR inhibition on cell functional properties. We first examined the effects of IGF-IR inhibition on an array of integrin dimer expression since several studies have demonstrated the cooperation of syndecan-4 with integrins^29^,^30^,^31^. Cell surface expression of four “RGD-binding” heterodimers α5β1, αvβ3, αvβ5, and αvβ6 was significantly down-regulated in AG1024-treated MCF-7 cells (approximately 80%, 60%, 80%, and 80%, respectively) compared to those of control cells (Fig. 4A). These results indicated that IGF-IR inhibition down-regulated important adhesion elements combined with syndecan-4 internalisation. Subunits of laminin-binding integrins (α3, α6, β4) were expressed at low levels. However, IGF-IR inhibition led to significant up-regulation of expression levels of α2 and β1 integrin subunits, which can combine as a well-known collagen binding receptor (Fig. 4B). As a consequence, IGF-IR inhibition alone did not affect cell adhesion to collagen I, but it significantly increased (ca 50%) cell adhesion of E2-treated cells compared to untreated cells, indicating a possible cross-talk between integrins, IGF-IR and E2-ERα signaling pathways (Fig. 4C). Moreover, functional blocking of the α2β1 integrin heterodimer by the specific P1E6 antibody resulted in the reduction of MCF-7 cells adherence on type I collagen. This inhibitory effect was further enhanced following inhibition of IGF-IR. Notably, IGF-IR inhibition leads to significant down-regulation of major adhesive molecules, like syndecan-4 and integrins, except for the collagen binding α2 and β1 integrin subunits. The over-expression of α2 and β1 subunits in relation with the significant reduction of the adhesion ability of the MCF-7 cells by blocking their action (Fig. 4D) highlights α2β1 as a critical mediator of cell adhesion. On the other hand, IGF-IR inhibition significantly induced (ca 30%) the invasive potential of ERα-positive MCF-7 cells into collagen I gels in an E2-independent manner (Fig. 4E) and the invasiveness of these cells is not dependent on the actions of MMPs (Fig. 4F). Taken together, these results indicated that the cross-talk of IGF-IR and E2-ERα pathways affect cell adhesion but not invasion, the latter being affected by IGF-IR alone.

### ERα is essential for the protective actions of IGF-IR in breast cancer cells

**Figure 5:**
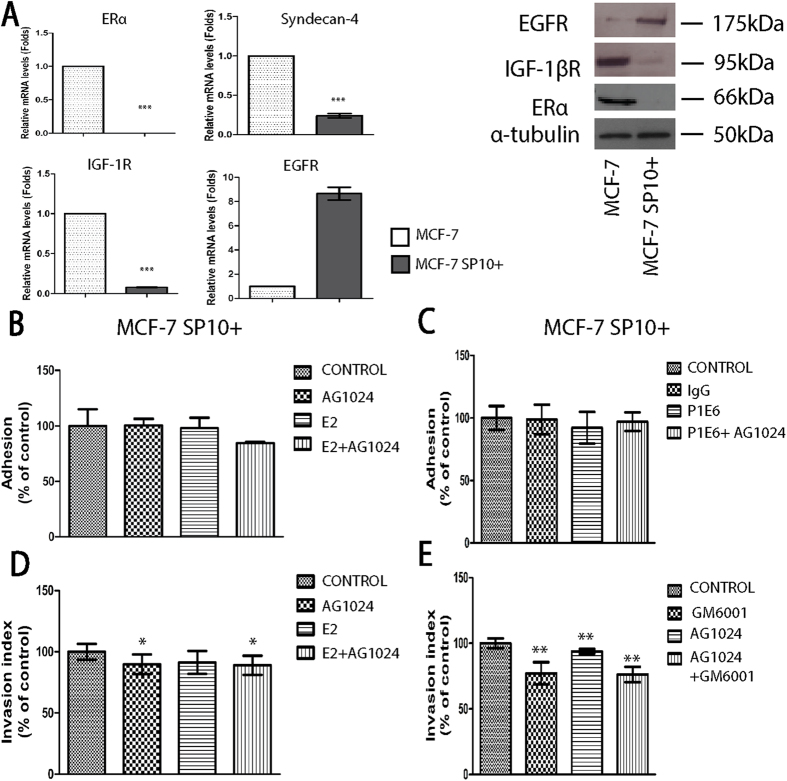
Loss of ERα strongly augments aggressiveness of MCF-7 breast cancer cells. (**A**) Confirmation of loss of ERα in MCF-7 SP10+ cells, which also led to almost complete loss of IGF-IR and syndecan-4 but to increased levels of EGFR. Gene expression levels were measured by Real-Time PCR and protein expression levels were measured by western blotting analysis. (**B**) The effect of IGF-IR on cell adhesion to collagen I substrate in the presence or absence of E2 of MCF-7 SP10+ breast cancer cells. (**C**) The blocking of α2β1 integrin had no effect on adhesion to collagen I of MCF-7 SP10+ cells. (**D**) The effect of IGF-IR on both the constitutive and the E2-mediated cell invasion on MCF-7 SP10+ breast cancer cells, respectively. Cell invasion was assessed by collagen type I assay. Cancer cells were incubated with the inhibitor of IGF-IR (AG1024, 1 μΜ) for 30 min, followed by the introduction of E2 (10 nM), where it was necessary. Total incubation time was 24 h. (**E**) The inhibition of MMPs, in the presence or the absence of IGF-IR inhibitor, led to suppression of invasiveness of MCF-7 SP10+ cells. Statistically significant differences compared with control or E2-treated cells are symbolized with *(p < 0.05), **(p < 0.01), ***(p < 0.001).

To further investigate the role of IGF-IR and its cross-talk with E2-ERα signaling pathway in the functional properties of ERα-positive breast cancer cells, the same experimental protocols were applied to ERα-knock down MCF-7 breast cancer cells (designated MCF-7/SP10+ cells) previously established in our laboratory^32^. Notably, these cells exhibited significantly lower mRNA and protein levels of IGF-IR and syndecan-4, while those of EGFR were significantly increased compared to the parental MCF-7 cells (Fig. 5A). Importantly, the strong stimulatory effect of IGF-IR inhibition on cell adhesion to collagen I in E2-treated ERα-positive MCF-7 cells was lost in ERα-knock down cells (MCF-7/SP10+ cells) revealing that ERα is essential for the suppressive action of IGF-IR in cell adhesion (Fig. 5B). Moreover, in ERα-knock down cells the α2 integrin subunit is not essential for the adherence of these cells compared to parental MCF-7 (Fig. 5C). Moreover, the stimulatory effect of IGF-IR inhibition on the invasiveness of ERα-positive MCF-7 cells was lost when ERα was knocked down (MCF-7/SP10+ cells) suggesting that ERα is also necessary for the suppressive action of IGF-IR on cell invasion (Fig. 5D). In contrast to MCF-7 cells, for ERα-knock down cells, MMPs seem to be crucial for their invasiveness (Fig. 5E). Taken together, these results demonstrate that IGF-IR reduces the aggressiveness of ERα-positive breast cancer cells and suggest that the protective role of IGF-IR and/or its cross-talk with ERα in these cells is lost in ERα-suppressed breast cancer cells.

## Discussion

Estrogens are pivotal in the growth of both normal and neoplastic mammary tissues and mediate most of their actions via estrogen receptors. Both genomic and non-genomic actions of E2 play crucial roles in E2-induced cancer cell proliferation and survival^33^. Since the majority of breast tumors are ER-dependent, blockade of E2 synthesis with aromatase inhibitors or antagonism of its action with anti-estrogens, represent first-line treatments for patients with ER-positive breast cancer. However, the majority of ERα-positive tumors, even if initially responsive to treatment with anti-estrogenic reagents such as tamoxifen, will eventually develop resistance to this treatment^34^. Membrane ERs may play a significant role in this resistance inducing the activation of key cell membrane receptors, such as IGF-IR and EGFR^35^,^36^. Both IGF-IR and EGFR are receptor tyrosine kinases with similar intracellular signaling pathways, including the activation of MAPKs or PI3K. Insulin-like growth factor-1 (IGF-I) is an important mediator of cellular proliferation and is strongly linked to the progression of a number of human cancers, including breast cancer. In this study, we provide data that support the significant role of IGF-IR in the aggressiveness of breast cancer cells. The significance of this receptor in cancer homeostasis is known. Specifically, the inhibition of the IGF-I/IGF-IR pathway results in reduced growth of breast cancer cell lines^37^. Moreover, IGF-IR is expressed at high levels in breast cancer, while its expression is positively correlated with estrogen receptor levels^38^. On the other hand, the EGFR is involved in various aspects of cell growth, survival, differentiation, migration, and invasion^39^,^40^. EGFR is often present in excessive amounts in human breast cancers. Moreover, several studies imply a cooperation of membrane ERs with IGF-IR and EGFR. More specifically, the mechanism of this cooperation is a linear activation of IGF-IR by ERs which in turn activates several MMPs. MMPs shed heparin binding-epidermal growth factor (HB-EGF) that binds the receptor resulting in the phosphorylation of EGFR, and downstream signaling cascades that include the MAP kinases^41^,^42^,^43^,^44^.

MMPs are key players in tumor progression because of their ability to remodel ECM and cleave/activate membrane-bound and pericellular growth factors, ECM proteins, and cytokines that stimulate cancer cell proliferation, migration and invasion^45^,^46^,^47^. A plethora of MMPs are over-expressed in breast tumors, though different MMPs mediate specific cell functions. For example, MMP-9 and MT1-MMP regulate breast cancer cell invasion and migration^48^,^49^,^50^,^51^. We have demonstrated previously that the action of E2 regulates gene expression and activity of MMPs^52^,^53^. In addition, the expression and the activity of MMPs/TIMPs may also be modulated by either IGF-IR or EGFR. EGF induces the expression of MMP-9 in SKB3 breast cancer cells^54^ while the introduction of exogenous IGF-I enhances MMP activity in MCF-7 breast cancer cells^55^. However, it is not yet known whether the interplay between these signaling axes may regulate the expression of MMPs/TIMPs in breast cancer^56^.

Here, we examined the importance of ECM in the cross-talk between ERs/IGF-IR/EGFR in breast cancer cell aggressiveness. In the case of MMPs, the membrane associated MT1-MMP was down-regulated in the presence of IGF-IR inhibitor, but in the additional presence of E2, which results in the activation of ERα, MT1-MMP returned to control levels. On the other hand, EGFR seems to act the other way around, with significant down-regulation of MT1-MMP levels in the presence of EGFR inhibitor or activation of IGF-IR with IGF. In the same system, MMP-9 as well as TIMP-1 and TIMP-2 have similar expression patterns to MT1-MMP. These data indicate that IGF-IR activation regulates critical proteolytic enzymes by balancing the expression levels of the proteases (MMPs) and their endogenous inhibitors (TIMPs). Overall, these data indicate that IGF-IR action drastically lowers the aggressive potential of breast cancer cells.

On the other hand, syndecans are molecules that are highly involved in cell adhesion and migration. In pathological conditions, increased syndecan shedding by proteinases like MMPs has been observed. Syndecan-4 promotes cell adhesion, in contrast to syndecan-1, which is increased during malignancies and promotes the aggressiveness of cancer cells^20^. After FACS analyses of cell surface syndecans levels in the presence or absence of RTKs (IGF-IR and EGFR) inhibitors, a significant down-regulation of syndecan-4, after treatment with IGF-IR inhibitor, in an E2-independent manner was observed. In the case of syndecan-1, only slight effects on protein levels after inhibition of IGF-R pathway were observed. Inhibition of IGF-IR resulted in a different localisation and cellular distribution of syndecan-4 and, to a lesser extent, syndecan-1. Syndecan-4 levels on the cell surface were decreased while stronger cytoplasmic staining was observed, consistent with the observed internalization of the proteoglycan. Immunoprecipitation of syndecan-4 identified IGF-IR in the same complex, indicating a potentially important synergistic role of IGF-IR on syndecan-4-mediated cell functions. It has been demonstrated previously that syndecan-1 and IGF-IR can be observed together in complexes^23^.

Syndecan-4 and integrins are key molecules in cell adhesion and migration on MCF-7 breast cancer cells and syndecan-4 reduced levels after inhibition of IGF-IR pathway could affect breast cancer cell behaviour. A significant reduction of the adhesive capacity caused by lower cell surface expression levels of α5β1, ανβ3, ανβ5 and ανβ6 integrins after inhibition of IGF-IR signaling pathway was observed. This result suggested that the inhibition of IGF-IR and the resulting endocytosis of syndecan-4 down-regulate the expression levels and/or stabilisation of other adhesive molecules like integrins from the cell surface, with the exception of the collagen binding α2β1 integrin, where a significant up-regulation was observed. The blocking of the α2β1 integrin heterodimer revealed that the up-regulated adhesion ability of MCF-7 cell on collagen type I substrate is mediated by α2β1 integrin. These data verify that adhesion of AG1024-treated MCF-7 cells was abolished on fibronectin substrates, but not collagen I because of the up-regulation of α2β1 integrin^57^. It is likely that larger molecular complexes containing syndecan-4, IGF-IR and integrins may exist. It is well known that integrins and syndecan-4 are concentrated in focal adhesions, for example^27^,^29^ and in the case of syndecan-1 the coupling with IGF-IR is responsible for integrin heterodimer activation^23^,^58^,^59^. It is therefore plausible that the inhibition of IGF-IR, with concomitant internalisation of syndecan-4, decreases the integrin(s) responsible for the adhesion of MCF-7 cells to fibronectin and laminin substrates, but not collagen type I, owing to α2 integrin overexpression. Moreover, decreased adhesion was associated with up-regulation of the invasive index after the inhibition of IGF-IR, in an E2- and MMP-independent manner, suggesting that the loss of adhesive molecules caused by IGF-IR inhibition has a dramatic effect on cell invasion-promoting ability of the MCF-7 cells.

The current data demonstrate that the co-operation of IGF-IR and E2-ERα signaling pathways is significant for breast cancer cell behaviour in promoting a non-aggressive phenotype. To independently examine this, we compared MCF-7 (ERα-positive) and MCF-7/SP10+ (ERα-suppressed) cell lines. Notably, MCF-7/SP10+ lacking ERα, but also IGF-IR and syndecan-4 as a result of the loss of ERα are characterised by a more aggressive phenotype and higher levels of EGFR compared to the parental MCF-7 cells. The inductive role of IGF-IR on cell adhesion is highly correlated with the expression levels of ERα, because the loss of ERα and IGF-IR led to less adherent cells and a striking increase in invasiveness. Moreover, the co-ordinated loss of ERα and IGF-IR led to significant reduction of syndecan-4 expression levels. In conclusion, the functions of ERα-positive and ERα-negative mammary carcinoma cells are regulated by different mechanisms; in ERα-positive cells, the IGF-IR and the adhesive molecules, like syndecans and integrins, play crucial roles, whereas in ERα-negative cells the MMPs mediate cancer cell invasion. These data underline the significant role of IGF-IR in the aggressiveness of breast cancer cells and correlate its action with ER status and syndecan-4 expression levels.

## Methods

### Chemicals, reagents and antibodies

Dulbecco’s modified eagles medium (DMEM), fetal bovine serum (FBS), sodium pyruvate, L-glutamine, penicillin, streptomycin, amphotericin B and gentamycin were all obtained from Biosera LTD (Courtabo euf Cedex, France). The cytostatic agent cytarabine, E2 the of EGFR inhibitor AG1478, the AG1024 inhibitor of IGF-IR, and EGF were also purchased from Sigma Chemical Co. (St Louis, MO, USA). IGF was purchased from R&D systems (Minneapolis, USA). All other chemicals used were of the best commercially available grade. Antibodies used were mouse monoclonal anti-syndecan-4 (5G9; Santa Cruz), mouse monoclonal anti-syndecan-1 (B-A38; Abcam), mouse monoclonal anti-HA (Clone HA.11; Covance), rabbit polyclonal against IGF-IRβ (D23H3; Cell signaling), rabbit polyclonal against ERα (HC-20, sc-534; Santa Cruz), rabbit polyclonal against ERβ (H-150, sc-8974; Santa Cruz) and mouse monoclonal to integrin alpha 2 + beta1 [P1E6] (ab24697; Abcam).

### Cell cultures

The MCF-7 (low metastatic, ERα-positive) breast cancer cell line was obtained from the American Type Culture Collection (ATCC) and routinely cultured as monolayers at 37 °C in a humidified atmosphere of 5% (v/v) CO_2_ and 95% air. MCF-7/SP10+ (ERα-suppressed cells) was previously described^32^. Breast cancer cells were cultured in DMEM supplemented with 10% (v/v) FBS, 1.0 mM sodium pyruvate, 2 mM L-glutamine and a cocktail of antimicrobial agents (0.8 μg/mL puromycin, 100 IU/mL penicillin, 100 μg/mL streptomycin, 10 μg/mL gentamycin sulphate and 2.5 μg/mL amphotericin B). Puromycin (0.8 μg/mL) was included in the cultures of MCF-7/SP10+ cells. Cells were harvested by trypsinization with 0.05% (w/v) trypsin in PBS containing 0.02% (w/v) Na_2_EDTA. Breast cancer cells were grown in serum-containing medium up to 80% confluence and then was followed overnight incubation in serum-free culture medium. The pre-treatment with AG1024 was for 30 min and then were added the E2 and growth factors for 16 h. All experiments were conducted in serum-free conditions. The transfection reagent for the performed experiments was the lipofectamine 2000 (Lipofectamine 2000, Invitrogen), which was used according to the manufacturer’s instructions.

### RNA isolation and real time PCR analysis

Cells were harvested and total RNA samples were isolated after cell lysis using NucleoSpin^®^ RNA II Kit (Macherey-Nagel, Duren, Germany). The amount of isolated RNA was quantified by measuring its absorbance at 260 nm.

Total RNA was reverse transcribed using the PrimeScript™ 1st strand cDNA synthesis kit (Takara Bio Inc., Japan) and KAPA TaqReadyMix DNA Polymerase (KAPABIOSYSTEMS). Real-time PCR analysis was conducted in 20 μL reaction mixture, consisting of 10 μL KAPA SYBR FAST Master Mix (2x) Universal (KAPABIOSYSTEMS) and 1 μΜ of template cDNA. The amplification was performed utilizing Rotor Gene Q (Qiagen, USA). Standard curves were run in each optimized assay which produced a linear plot of threshold cycle (Ct) against log (dilution). The amount of each target was quantified based on the concentration of the standard curve and was presented as arbitrary units. The quantity of each target was normalized against the quantity of GAPDH. Genes of interest and utilized primers are presented in Supplementary Table S1.

### Flow Cytometry

Cells were incubated with dissociation buffer (Invitrogen) and suspended in ice-cold PBS contains 1% FCS and stained with syndecan-1 (1:50) or syndecan-4 (1:50) antibodies for 30 min on ice. Then, the sample were washed three-times with FACS buffer and incubated with Alexa Flour-conjugated anti-mouse IgG for 30 min and analyzed on a FACSCalibur flow cytometer and the data were processed by using Cell Quest Pro v6.0 software (Becton Dickinson).

### Confocal Microscopy

Cells were seeded onto coverslips in 24-well plate and incubated in DMEM + 10% FCS for 24 h before transfection. After transfection cells were incubated for 48 h and then fixed in 4% paraformaldehyde in PBS and permeabilised with 0.1% Triton X-100 for 10 min. Coverslips were incubated with 0.1 M NH_4_Cl for 20 min to quench free aldehydes and blocked with 5% normal goat serum for 30 min. Afterwards, cells were stained with primary antibodies overnight at 4 °C and followed by incubation with Alexa Fluor-conjugated antibodies for 1 h at room temperature. Cells were mounted in Prolong Gold mounting media (Invitrogen), analyzed by confocal laser-scanning microscope (LSM510 Meta, Carl Zeiss, Oberkochen, Germany) equipped with a 63x/1.4 Plan-Apochromat oil immersion objectives and images processed using Zen Software.

### Adhesion assays

For measurements of cell surface integrin levels wells were coated with mouse monoclonal antibodies against integrin extracellular domains (Chemicon- Millipore) QCM™, ECM535). Monoclonal antibodies against α1, α2, α3, α4, α5, αV, αVβ3, β1, β2, β3, β4, β6, αVβ5, α5β1 integrins and a goat anti-mouse IgG as negative control were immobilised. Cells were cultured for 24 h, in the presence or absence of the IGF-IR inhibitor (1 μM AG1024). Cells were washed three times with a serum free medium and then suspended in serum free medium and 1.5 × 10^5^ cells were placed in the wells. After 2 h incubation the cells were washed three times with serum free DMEM so that non-adherent cells were removed. Afterwards the attached cells were lysed and incubated with CyQUANT ™ GR Dye. Measurements were made on a fluorimeter (Infinite^®^, Tecan) with excitation at 485 nm and emission at 530 nm.

In order to evaluate the adhesive potential of breast cancer cells the following adhesion protocol was conducted as described previously^60^. Briefly, 40 μg/ml of collagen type I in PBS and 0.1% BSA solution in serum free medium were prepared. 96-well plate coated for 12 h at 4 °C with collagen type I solution (30 μg/ml). Then, the solution was removed and the plate was air-dried. Cancer cells were deprived of serum for 8 h prior to the adhesion assay and in the case of blocking with P1E6 antibody or anti-mouse IgG, a 30 min incubation before the beginning of the assay in final concentration of 20 μg/mL for both antibodies was used. Then the cells were detached with PBS-EDTA 1x, re-suspended with 0.1% BSA and seeded at a density of 2 × 10^4^ cells/well. Cells were incubated for 30 min in order to be allowed to adhere to the surface. Non-adherent cells were removed with serum free medium and then cells were incubated with medium supplemented with 10% FBS for 4 h for recovery. After incubation period, Premix WST-1 (water-soluble tetrazolium salt) Cell Proliferation Assay System (Takara Bio Inc., Japan) was added at a ratio 1:10 and the absorbance at 450 nm was measured (reference wavelength at 650 nm).

### Western blotting and co-immunoprecipitation

Cells were lysed in a buffer containing 62.5 mM Tris-HCl pH 6.8, 2% sodium dodecyl sulfate (SDS), 10% glycerol, 5% β-mercaptoethanol and 0.001% bromophenol blue. Cell lysates were separated on 10% SDS-PAGE, the proteins were electrophoretically transferred to membranes PVDF (Bio-Rad, USA) and blotted with the indicated antibodies as previously^61^.

For the co-immunoprecipitation experiments, the breast cancer cells were transfected with plasmid encoding wild-type syndecan-4^61^ and cultured for 24 h. Afterwards, the cells were lysed in ice cold buffer containing 20 mM HEPES pH 7.5, 150 mM NaCl, 1% Triton- X100, 2 mM EDTA, 1 mM PMSF and protease inhibitor cocktail. Lysates were sheared through a 25 G needle and mixed for 1 hour at 4 °C. Lysates were centrifuged at 13,000 rpm for 5 min at 4 °C and supernatants were pre-adsorbed with protein G agarose beads (Sigma-Aldrich, St Louis, MO, USA) for 1 hour at 4 °C. The treated lysates were incubated overnight at 4 °C with anti-HA antibody and then incubated with protein G agarose beads for 1 hour at 4 °C. The beads were washed extensively followed by electrophoresis and immunoblotting analysis.

### Collagen invasion assay

The invasive potential of breast cancer cells was evaluated using the collagen type I invasion assay, as described previously^62^. In brief, collagen type I solution with final concentration of 1 mg/ml was prepared by mixing the pre-cooled components: 4 volumes collagen type I (stock concentration 3 mg/ml), 5 volumes of CMF-HBSS, 1 volume of DMEM (10x), 1 volume of 0.25 M NaHCO_3_, 2.65 volumes of DMEM and 0.3 volumes of 1 M NaOH. The solution was gently mixed and added to one well of 12-well plate, spread homogeneously and allowed to gel in a humidified atmosphere of 10% CO_2_ at 37 °C for >1 h. Breast cancer cells previously cultured in the presence or the absence of AG1024, E2, E2 + AG1024 and GM6001 in serum-free conditions for 16 h were seeded at a density of 6 × 10^4^ cells per well on the top of the collagen gels. Cells were incubated in a humidified atmosphere of 10% CO_2_ at 37 °C and after 24 h digital images were obtained with a 10x objective. The calculation of cell invasion was as described by De Wever *et al*.^62^.

### Statistical analysis

Reported values are expressed as mean  ±  standard deviation (SD) of experiments in triplicate. Statistically significant differences were evaluated using the analysis of variance (ANOVA) test and were considered statistically significant at the level of at least p ≤ 0.05. Statistical analysis and graphs were made using GraphPad Prism 6 (GraphPad Software).

## Additional Information

**How to cite this article:** Afratis, N. A. *et al*. IGF-IR cooperates with ERα to inhibit breast cancer cell aggressiveness by regulating the expression and localisation of ECM molecules. *Sci. Rep.* **7**, 40138; doi: 10.1038/srep40138 (2017).

**Publisher's note:** Springer Nature remains neutral with regard to jurisdictional claims in published maps and institutional affiliations.

## Supplementary Material

Supplementary Table
