# Supplementary material for: IGF-IR cooperates with ERα to inhibit breast cancer cell aggressiveness by regulating the expression and localisation of ECM molecules
Source: Sci Rep. 2017 Jan 12;7:40138. doi: 10.1038/srep40138 (PMC5228153; doi:10.1038/srep40138)
Supplement: Supplementary Table [file srep40138-s1.pdf]

IGF-IR cooperates with ERα to inhibit breast cancer cell aggressiveness by regulating the expression and localization of ECM molecules

Nikolaos A. Afratis [1,2#], Panagiotis Bouris [1#], Spyros S. Skandalis [1], Hinke A. Multhaupt [2], John R. Couchman [2], Achilleas D. Theocharis [1,*] and Nikos K. Karamanos [1,*]

[1] Biochemistry, Biochemical Analysis & Matrix Pathobiology Res. Group, Laboratory of Biochemistry, Department of Chemistry, University of Patras, Patras 26110, Greece;
[2] Biotech Research and Innovation Center, University of Copenhagen, Ole Maaløes Vej 5, 2200 Copenhagen N, Denmark.

*Correspondence to Nikos K. Karamanos: Laboratory of Biochemistry, Department of Chemistry, University of Patras, 26110 Patras, Greece. n.k.karamanos@upatras.gr and Dr. Achilleas D. Theocharis, address as above. atheoch@upatras.gr

[#]These authors contributed equally

Table 1. Sequence of the primers used for Real Time PCR analysis

**Gene of interes**		**Primer sequence**	**Annealing Temperature ($T_{annealing}$) ^{0}C**
MMP-9	F	TTC CAG TAC CGA GAG AAA GCC TAT	60
	R	GGT CAC GTA GCC CAC TTG GT	
MT1-MMP	F	CAT GGG CAG CGA TGA AGT CT	60
	R	CCA GTA TTT GTT CCC CTT GTA GAA GTA	
TIMP-1	F	CGC TGA CAT CCG GTT CGT	60
	R	TGT GGA AGT ATC CGC AGA CAC T	
TIMP-2	F	GGG CAC CAG GCC AAG TT	60
	R	CGC ACA GGA GCC ATC ACT	
GAPDH	F	AGG CTG TTG TCA TAC TTC TCA T	60
	R	GGA GTC CAC TGG CGT CTT	
	R	GGG TCC ATT TTC CTT TCT GAG T	
Syndecan-4	F	GTG TCC AAC AAG GTG TCA ATG T	60
	R	CGG TAC ATG AGC AGT AGG ATC A	
